# Outcomes of Complex Circumcisions for Pre-cancerous and Cancerous Penile Lesions at a Tertiary Referral Centre: A Retrospective Cohort Study

**DOI:** 10.7759/cureus.89893

**Published:** 2025-08-12

**Authors:** Mahmoud Elmousili, Abdul Hadi Kafagi, Muhammad Abdullah, Panagiotis Christopoulos, Maurice Lau, Arie Parnham, Vijay Sangar

**Affiliations:** 1 Department of Urology, Wythenshawe Hospital, Manchester University NHS Foundation Trust, Manchester, GBR; 2 Department of Urology, Faculty of Biology, Medicine and Health, The University of Manchester, Manchester, GBR; 3 Department of Urology, The Christie Hospital, The Christie NHS Foundation Trust, Manchester, GBR

**Keywords:** circumcision complications, clavien-dindo classification, complex circumcision, lichen sclerosus, male circumcision, penile cancer, penile intra-epithelial neoplasia (pein), penile intraepithelial neoplasia (pein), post-operative complications, retrospective studies

## Abstract

Objective: This study aimed to evaluate the complication rates of complex circumcisions performed for penile cancer, penile intra-epithelial neoplasia (PeIN) and severe lichen sclerosus, and to identify any predictors of post-operative complications.

Patients and methods: A retrospective cohort study was conducted at a tertiary urology referral centre. Records of 191 male patients who underwent complex circumcisions between 2014 and 2020 were reviewed. Complex circumcision was defined as circumcision for cancerous or pre-cancerous lesions, condyloma or severe lichen sclerosus with >50% adherence to the glans. Patient demographics, indications, surgical variables and complications were analysed. Complications were graded using the Clavien-Dindo classification.

Results: The overall complication rate was 11%. The most common complications were infection (n = 5) and altered glans sensation (n = 5). Consultant-performed procedures had a significantly higher complication rate (14%) compared to those performed by trainees (2.3%) (p = 0.05), likely reflecting case complexity. No statistically significant associations were found between complications and patient age, indication, cancer stage or grade, type of circumcision or suture technique. All complications were Clavien-Dindo Grade I or II, and there were no readmissions.

Conclusion: Complex adult circumcisions carry a modest complication risk, particularly in oncological cases. While consultant-led procedures had higher complication rates, this likely reflects the referral of more complex pathology. These findings provide a UK-based evidence base for consenting patients undergoing circumcision for malignant or pre-malignant indications.

## Introduction

Circumcision is the most widely performed urological procedure globally and is carried out for a variety of religious, cultural and medical reasons [1]. In adults, circumcision is commonly indicated for conditions such as phimosis, recurrent balanitis and Lichen sclerosus (also referred to as balanitis xerotica obliterans or BXO) [2]. Adult circumcision plays an important role not only in symptom relief but also as a part of oncological management in malignant and pre-malignant foreskin diseases [3].

Complex circumcisions refer to procedures performed in the context of significant penile pathology, such as penile cancer, penile intra-epithelial neoplasia (PeIN), condyloma or advanced lichen sclerosus with adhesions between the foreskin and glans. These procedures are more technically challenging and are frequently performed by specialist surgeons at tertiary referral centres. The surgical complexity, distorted anatomy and presence of diseased tissue in these cases increase the risk of post-operative complications compared to standard or cosmetic circumcisions [4].

Although circumcision is common in urological practice, most research focuses on newborns, children or adults who undergo the procedure for cosmetic or minor medical reasons. These studies may not apply to more complex adult circumcisions, especially those done to treat penile cancer. There is a lack of research on the outcomes and complications of complex circumcisions, particularly in the United Kingdom. More studies are needed to better understand the risks and recovery from these surgeries [5].

Accurate knowledge of post-operative risks is essential for effective patient counselling and shared decision-making [6]. Despite its clinical relevance, there is a paucity of literature specifically addressing outcomes related to complex circumcisions performed for oncological or severe inflammatory indications. Most existing studies focus on routine circumcision, with limited attention paid to this subset of patients who present with high-risk pathology. Data are particularly scarce regarding surgical outcomes, complication rates and the diagnostic yield of circumcision in the evaluation of penile malignancy.

This study aims to characterise patients undergoing complex circumcisions at a tertiary referral centre in the United Kingdom. These procedures are technically demanding and often require advanced surgical skills. As a result, they are typically performed by specialist surgeons in tertiary referral centres. By examining indications, surgical approaches, operator experience, post-operative complications and histological outcomes, we aim to provide a comprehensive overview of the role and safety of complex circumcision in the management of high-risk penile pathology.

## Materials and methods

This study was a retrospective nonconcurrent cohort analysis conducted at a tertiary referral urology centre in the United Kingdom. It included adult male patients who underwent complex circumcision between January 2014 and December 2020. Before data collection, the study was registered with the institutional audit committee.

Complex circumcisions were defined as those performed for oncological or pre-oncological indications, including squamous cell carcinoma of the penis (SCCP), PeIN, condyloma acuminata or severe lichen sclerosus with more than 50% foreskin adherence to the glans [7]. Cases of revision circumcision for recurrent or residual malignancy were also included.

Patients were identified using surgical logs and operation theatre records. Inclusion criteria were age >18 years, documented diagnosis meeting the definition of complex circumcision and availability of complete medical records, including operative and follow-up notes. Patients were excluded if the procedure was performed for non-complex indications (e.g., cosmetic or routine phimosis) or if records were incomplete.

All procedures were performed under general anaesthesia. While the surgical approach was not formally standardised, all procedures utilised a sleeve excision technique [8,9] with or without a dorsal slit, in line with standard departmental practice. Surgeons included consultant urologists, specialist registrars and clinical fellows. Suture closure (one-layer vs. two-layer) was based on individual surgeon preference and documented where available.

Data were extracted by a single reviewer (M.A.) using the hospital’s electronic patient record system (Clinical Web Portal). The data collected included patient age, surgical indication, surgeon grade, type of circumcision (standard, redo, radical), presence of additional procedures, suture technique and post-operative complications. Where cancer was the indication, histopathological stage and grade were also recorded. Follow-up notes were reviewed for documentation of complications.

Patients with confirmed cancerous pathology were routinely followed up in the clinic at four weeks post-operatively. Other patients were typically discharged following review at the surgical team's discretion. As this was a retrospective review, follow-up length and depth varied depending on the clinical pathway.

The primary outcome was the occurrence of post-operative complications, classified using the Clavien-Dindo grading system (Table 1) [10]. This included infection, altered glans sensation, erectile dysfunction, wound breakdown, glans dryness, cosmetic dissatisfaction, skin tightness and pain. Secondary outcomes included the nature and severity of complications, as well as associations between complications and clinical and surgical variables.

**Table 1:** Summary of Clavien-Dindo classification of surgical complications Source: [10]

Grade	Details
1	Deviation from the normal post-operative course, but without any need for pharmaceutical treatment or surgical, endoscopic and radiological interventions. Acceptable drugs are antiemetics, antipyretics, analgesics, diuretics, electrolytes and physiotherapy. Also includes wounds open at bedside due to infection
2	Require pharmaceutical treatments with drugs not used in grade 1 complications. Include antibiotics, blood transfusions and total parenteral nutrition alongside other drugs
3	Require a surgical, radiological or endoscopic intervention
3a	Need an intervention under local/regional anaesthesia
3b	Need an intervention under general anaesthesia
4	Life-threatening complications that need ICU-level care
4a	Single-organ dysfunction
4b	Multi-organ dysfunction
5	Death of a patient

Data were entered into Microsoft Excel (Microsoft Corporation, Redmond, WA) and analysed using Statistical Package for the Social Sciences version 25 (IBM Corp., Armonk, NY; 2018). Descriptive statistics were used for patient demographics and complication rates. Due to small sample sizes, Fisher’s exact test was employed to determine associations between categorical variables and complications. An independent t-test was used to compare the mean age between the groups with and without complications. Statistical significance was defined as p < 0.05. Cramer’s V was used to assess the strength of associations where appropriate.

## Results

A total of 191 patients underwent complex circumcision and were included in the analysis. The median age was 62 years (range 21-92). Surgical indications included penile cancer or suspicious lesions (n = 81), PeIN (n = 39), lichen sclerosus or BXO (n = 24), severe phimosis or other benign causes (n = 35), and condyloma acuminata (n = 2).

**Figure 1:**
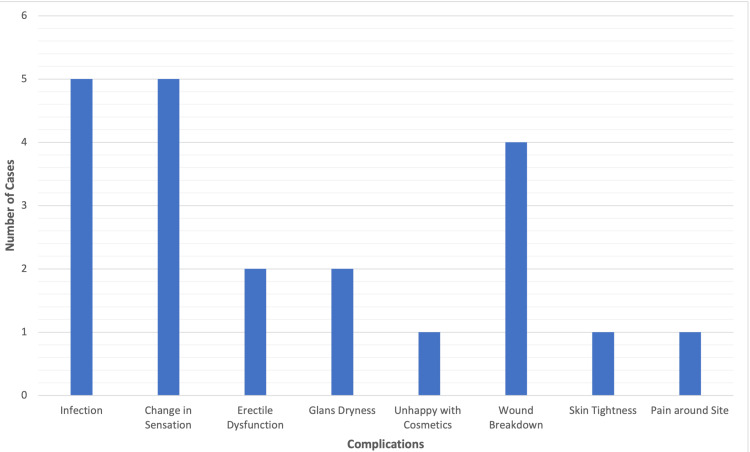
Complications observed in 21 cases, with infection and altered glans sensation being the most common

Post-operative complications were observed in 21 patients, corresponding to an overall complication rate of 11%. The most frequent complications were post-operative infection (n = 5, 2.6%) and altered glans sensation (n = 5, 2.6%). Other complications included wound breakdown (n = 4, 2.1%), erectile dysfunction (n = 2, 1.0%), glans dryness (n = 2, 1.0%), cosmetic dissatisfaction (n = 1, 0.5%), skin tightness (n = 1, 0.5%) and localised pain (n = 1, 0.5%) (Figure 1). No major complications (Clavien-Dindo Grade III or higher) were reported, and no patients required re-admission or further surgical intervention.

Complications by indication

**Figure 2:**
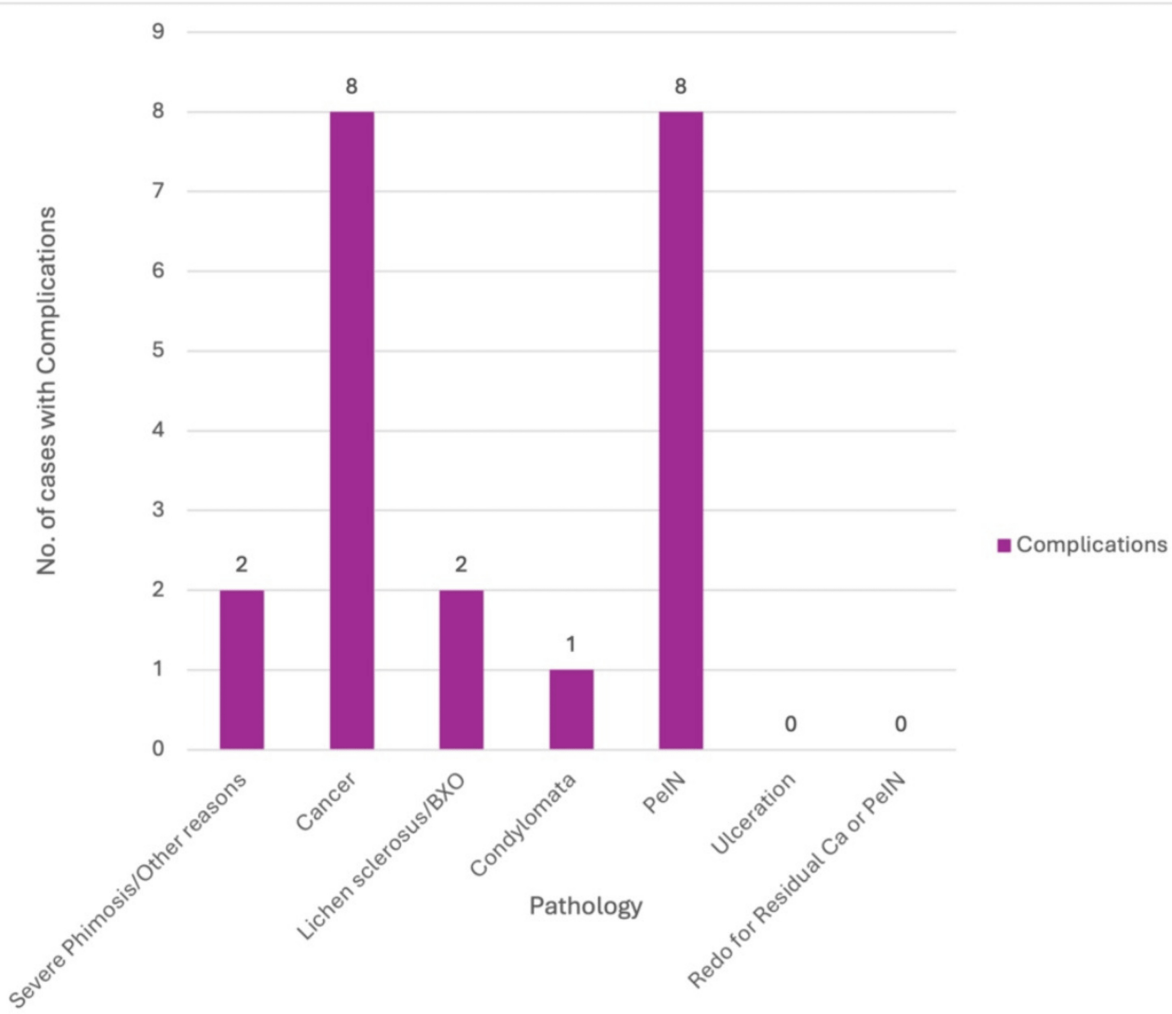
Pathologies associated with post-circumcision complications. Most occurred in cancer and PeIN cases; none in ulceration or redo procedures BXO: balanitis xerotica obliterans; PeIN: penile intra-epithelial neoplasia

Cancer and PeIN were the most common indications among patients who developed complications (n = 8 each). Lichen sclerosus was associated with two complications, and severe phimosis with two. A single complication was observed in a patient with condyloma (Figure 2). Statistical analysis revealed no significant association between the underlying pathology and the incidence of complications (Fisher’s exact p = 0.225). Cramer’s V analysis demonstrated a weak association (V = 0.220).

Complications by operator grade

Consultants performed 147 procedures (77%), and trainees (specialist registrars or clinical fellows) performed 44 (23%). Of the 21 complications, 20 occurred in consultant-led procedures (14% complication rate), while only one occurred in a trainee-led procedure (2.3%). This difference was statistically significant (Fisher’s exact p = 0.05), though the association was weak (Cramer’s V = 0.149), suggesting case complexity as a likely confounder.

Cancer characteristics and complications

Among 63 cases with confirmed SCCP on histology, complications were most frequently observed in patients with stage T1a or T1b disease. No complications were observed in T2 cases. Cancer stage and grade did not significantly correlate with complication risk (stage: p = 0.786; grade: p = 0.727). Cramer’s V values for both analyses indicated weak associations (0.119 and 0.145, respectively).

Type of circumcision and complications

Three surgical types were recorded: standard circumcision (n = 104), redo circumcision (n = 23), and radical circumcision (n = 64) (Table 2). Most complications occurred following standard circumcision (n = 13), with fewer in radical (n = 5) and redo procedures (n = 3). No significant association was found between the type of circumcision and complication incidence (p = 0.606, Cramer’s V = 0.072).

**Table 2:** Summary of the number of cases with and without complications for each type of circumcision

Type of circumcision	Details	Complications	No complications	Total circumcision
Standard	Some foreskin is left on the penis	13	91	104
Re-do	Performed on patients who already had a circumcision once	3	20	23
Radical	No foreskin is left, and the skin of the shaft of the penis is attached directly to the glans	5	59	64

Age and complications

**Figure 3:**
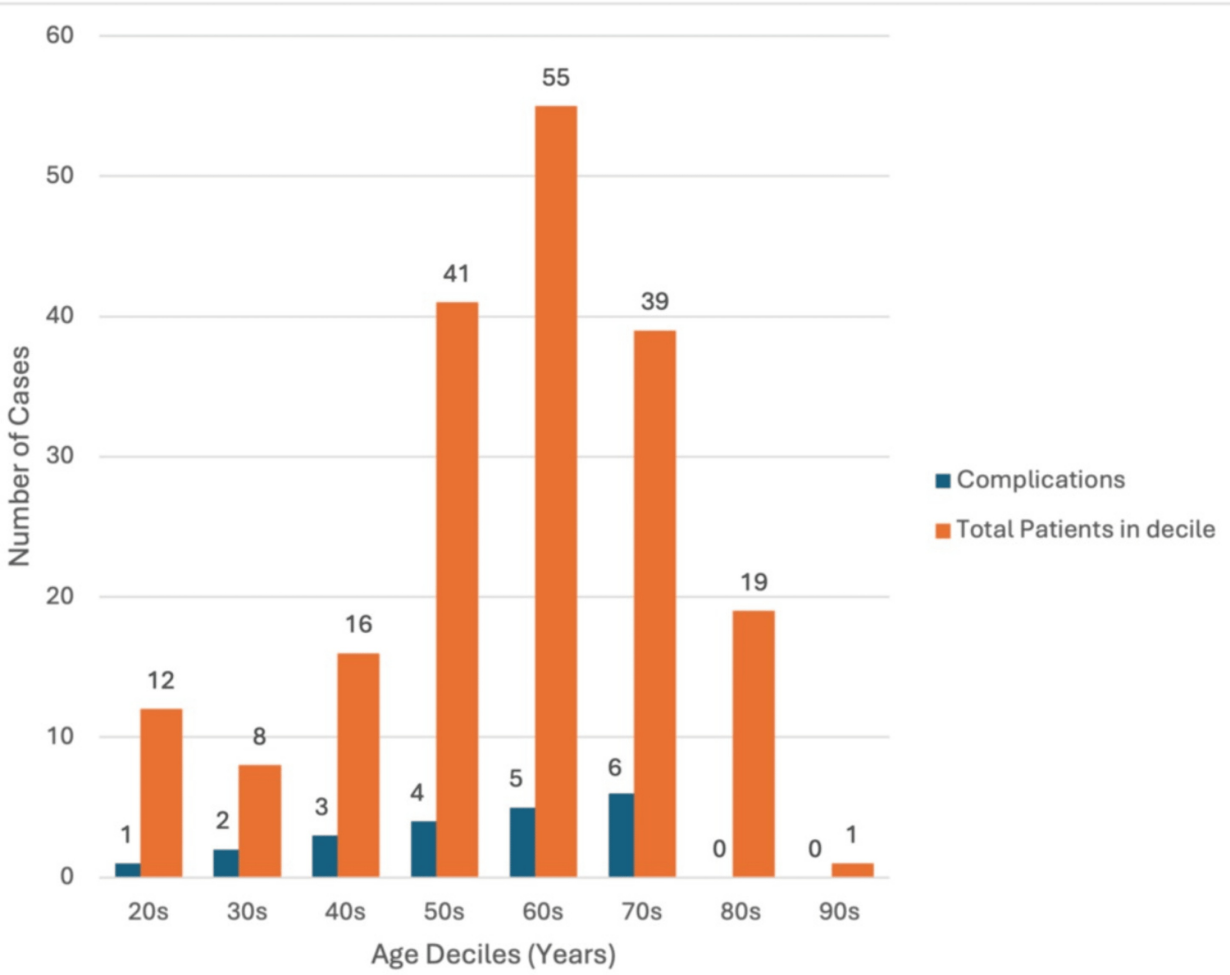
Complications by age decile. Most procedures occurred in patients in their 60s, with the highest complication rate in those in their 70s

The mean age of patients who developed complications was 58.1 years (SD ±14.8), compared with 61.3 years (SD ±15.4) in those who did not (Figure 3). This difference was not statistically significant (t = 0.901, p = 0.369). No significant association with complication rate was found when categorised as <60 years or ≥60 years (p = 0.488).

Suture technique and complications

The closure technique was documented in 147 cases. Of these, 74 were closed with a single layer and 73 with two layers. The incidence of complications was evenly distributed (n = 8 and n = 7, respectively), with no significant association observed (Fisher’s exact p = 1.00, Cramer’s V = 0.020).

Additional procedures and complications

Additional surgical procedures were performed in 50 cases. The most common were wide local excision (WLE, n = 44) and biopsy (n = 25). Complications were observed most frequently in patients undergoing WLE (n = 8) and in those without additional procedures (n = 11). No statistically significant correlation was found (p = 0.056), though Cramer’s V (0.237) suggested a weak association.

Severity of complications

**Figure 4:**
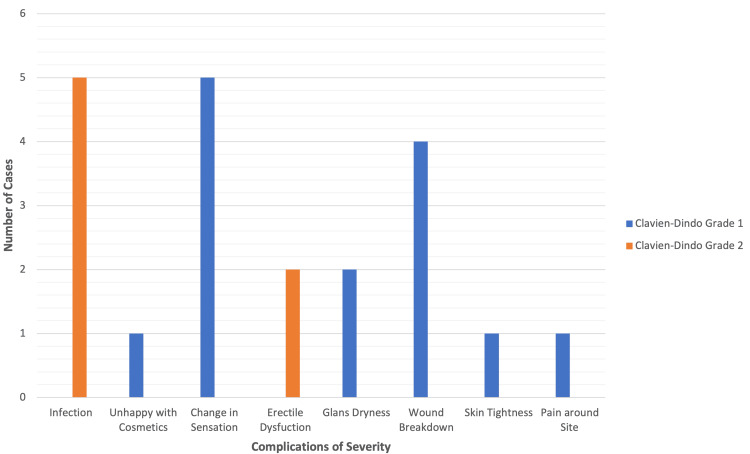
Severity of complications by the Clavien-Dindo grade. Only infection and erectile dysfunction were grade 2; all others were grade 1 Source: [10]

All complications were Clavien-Dindo Grade I or II (Figure 4 and Table 1). Infections and erectile dysfunction were classified as Grade II due to the need for pharmacological intervention. All others were Grade I. No Grade III-V complications were recorded, and no patients required reoperation or intensive care.

Readmissions

There were no hospital readmissions within 30 days after the procedure.

## Discussion

The findings from this study suggest that while complex adult circumcision is generally safe, its complication profile differs in subtle but clinically meaningful ways from that of standard or cosmetic circumcision. This distinction has important implications for how we approach perioperative counselling and consent in patients undergoing circumcision for oncological or pre-malignant conditions.

Infection was the most reported complication in our cohort, occurring in 2.6% of cases. This figure aligns with the upper end of infection rates reported in standard circumcision literature, which typically ranges from 0.4% to 2% according to the British Association of Urological Surgeons (BAUS) guidelines [11]. Similarly, the American Academy of Paediatrics and several large cohort studies report that infection following circumcision occurs in fewer than 1% of cases [12]. This modestly elevated rate in our complex cohort may reflect both the inherent challenges of operating on diseased tissue and the increased risk posed by pre-existing inflammation, tissue adherence or prior surgical interventions. Notably, while the infections observed were mild (Clavien-Dindo Grade I or II) and self-limiting, their presence nonetheless impacts recovery time and patient satisfaction.

Altered glans sensation was also noted in 2.6% of patients in our study. Interestingly, this is significantly lower than what is described in standard circumcision literature, where BAUS suggests that altered sensation may occur in nearly all patients to some extent [11]. The lower incidence in our series could represent underreporting, especially as patients undergoing surgery for malignancy may be less focused on sensory changes than those seeking cosmetic procedures. Alternatively, it may be that in complex cases, where glandular desensitisation may already be present due to chronic inflammation or scarring, additional sensory changes are not perceived as a significant deviation from baseline [13].

Wound breakdown was observed in 2.1% of patients in our cohort. This again exceeds the 1.2% wound dehiscence rate reported in a multi-centre randomised controlled trial by Azizoglu et al. [14], which compared Alisklamp and conventional dorsal slit circumcision in adult patients. Their study, involving a large sample of adult males, provides a relevant comparator for standard circumcision techniques. The increased incidence in our cohort likely reflects the more complex nature of the pathology, including malignancy and severe lichen sclerosus, which often necessitate more extensive dissection. Additional contributing factors include the presence of fibrotic or fragile tissue and concurrent procedures such as WLE. These elements may compromise wound integrity and impair healing, thereby increasing the likelihood of dehiscence in this surgical subgroup.

Erectile dysfunction occurred in 1% of patients in our study. While most studies on standard circumcision suggest that there is no significant long-term impact on erectile function, the literature remains divided. Fink et al. demonstrated that adult circumcision was associated with worsened erectile function (p = 0.01) and decreased penile sensitivity (p = 0.08), based on a paired pre- and post-circumcision analysis [15]. However, establishing causality remains challenging due to confounding factors such as age, psychological state and comorbidities. In our series, both patients who reported erectile dysfunction were over the age of 50, though detailed evaluation of baseline function or contributory factors was not undertaken. While rare, the occurrence of erectile dysfunction in this context is not negligible and should be explored further in larger prospective studies.

Other complications, such as glans dryness (1.0%), cosmetic dissatisfaction (0.5%), skin tightness (0.5%) and pain (0.5%), were all infrequently reported. In standard circumcision, these complications are also relatively uncommon [6], though dissatisfaction with cosmetic outcome can be more prominent in cosmetic procedures. Our low rates of dissatisfaction may reflect the therapeutic nature of the procedure in our cohort, where patients were more concerned with disease control than cosmetic appearance. The context of surgery plays a key role in shaping patient expectations, and this likely mitigates some of the psychosocial distress that might otherwise arise from minor aesthetic or sensory changes. These findings are further contextualised in Table 3, which compares complication rates observed in our study with those reported in existing literature and clinical guidelines.

**Table 3:** Comparison of post-operative complication rates in complex circumcision vs. existing literature and guidelines BAUS: British Association of Urological Surgeons

Complication type	Our study (n = 191)	BAUS leaflet [11]	Shabanzadeh et al. [5]	Fink et al. [15]	Azizoglu et al. [14]
Infection	2.6%	0.4-2%	~1.5% total complications; 0.5% infection	Not reported	Not specified
Altered glans sensation	2.6%	Common (qualitative)	Not reported	Sensitivity ↓ in some	Not specified
Wound breakdown	2.1%	Not specified	Not specified	Not reported	1.2%
Erectile dysfunction	1%	Not specified	Not specified	Reported ↓ in erection in some	Not reported
Glans dryness	1%	Described qualitatively	Not reported	Not reported	Not reported
Cosmetic dissatisfaction	0.5%	~0.4-2%	Not specified	Satisfaction generally ↑	Low, not quantified
Skin tightness	0.5%	Not specified	Not specified	Not reported	Not reported
Pain	0.5%	Mentioned transiently	Not specified	Not reported	Low, not quantified

When comparing these outcomes with those of standard circumcision, complex circumcisions carry a slightly elevated risk in certain areas, particularly infection and wound-related complications. However, the absolute increase in risk is modest, and all complications in this cohort were low-grade and manageable without re-admission or surgical re-intervention.

These findings carry important implications for informed consent. In patients undergoing circumcision for malignant or pre-malignant lesions, clinicians should take care to explain the modestly increased risk of infection, wound healing issues and sensory changes. While these risks do not appear to compromise the procedure's overall safety profile significantly, they may influence patient expectations and post-operative satisfaction. Furthermore, the potential for sexual dysfunction, though rare, should be discussed, particularly in older patients or those undergoing extensive resection.

In summary, while complex circumcision is broadly safe, it is distinct from standard circumcision in both indication and risk profile. Surgeons should be mindful of these differences and ensure that counselling is appropriately tailored to the clinical context. This includes framing the procedure within the goals of disease management, while transparently communicating the elevated risks associated with more extensive pathology and surgical dissection.

Bias and limitations

This study is subject to limitations typical of retrospective reviews, including reliance on accurate documentation and lack of data on patient comorbidities such as diabetes or smoking status, which may influence complication risk. Complication reporting was clinician-dependent, and no standardised patient-reported outcomes were available. Data were extracted by a single reviewer without independent validation, which may introduce selection or reporting bias. However, all efforts were made to ensure complete and consistent data extraction.

## Conclusions

Complex adult circumcision is a safe procedure with a low complication rate, even in high-risk oncological cases. These findings support the need for thorough pre-operative counselling and suggest that complication risk is not significantly influenced by patient age, surgical technique or cancer characteristics. Prospective multi-centre studies are warranted to further evaluate predictors of complications and improve patient selection and consent processes.
